# Adherent expansion of CHO-K1 and HEK-293 cells in a rotating polylactic-acid bioreactor: a scalable bioprocess^,^

**DOI:** 10.1016/j.mex.2026.104147

**Published:** 2026-09-06

**Authors:** Fabian Watzl, Johanna Eichberg, Annika Herchenröder, Katharina Günther, Lukas Käßer, Björn Boshof

**Affiliations:** Green Elephant Biotech GmbH, Kerkrader Straße 9, Gießen, 35394, Germany

**Keywords:** Adherent cell culture, CHO-K1, HEK-293, Cell expansion, Cell Screw®, Polylactic acid, Sustainable process scale-up

## Abstract

Adherent cell expansion at intermediate scale (<10,000 cm²) typically relies on parallelized T-flasks, roller bottles, multilayer stacks, fixed-bed bioreactors, or microcarriers, each involving trade-offs in handling complexity, shear stress, medium economy, or plastic waste. The CellScrew® addresses these limitations by replacing multiple petroleum-derived vessels with one polylactic acid (PLA) vessel. During rotation, its concentric cylinders arranged in an Archimedean screw generate a fluid film distributing medium and dissolved gases under low-shear while cells remain attached. This substantially reduces incubator footprint: one shelf accommodates three CellScrew® 6Ks compared with 36 T175s, roughly tripling growth area while lowering material use and handling time.

We describe a reproducible protocol for CHO-K1 and HEK-293 expansion in the 850 cm² and 6,000 cm² formats, covering inoculation from T-flask seed trains, in-process supernatant monitoring, and harvest. Quantified attachment, proliferation kinetics, harvest efficiency, and post-thaw recovery showed consistent performance and transferability to other adherent cells.

• Expansion standardized in both formats, reaching 200,000 cells/cm² (CHO-K1; 850 cm²) and 361,000 cells/cm² (HEK-293; 6,000 cm²).
• Mechanical-enzymatic harvest recovers 94 ± 1% of cells at >93% viability.
• HEK-293 attachment within first 24 h from polystyrene without multi-passage adaptation.
• In-process metabolite monitoring as an indirect readout of culture density.

## Graphical abstract

**Figure:**
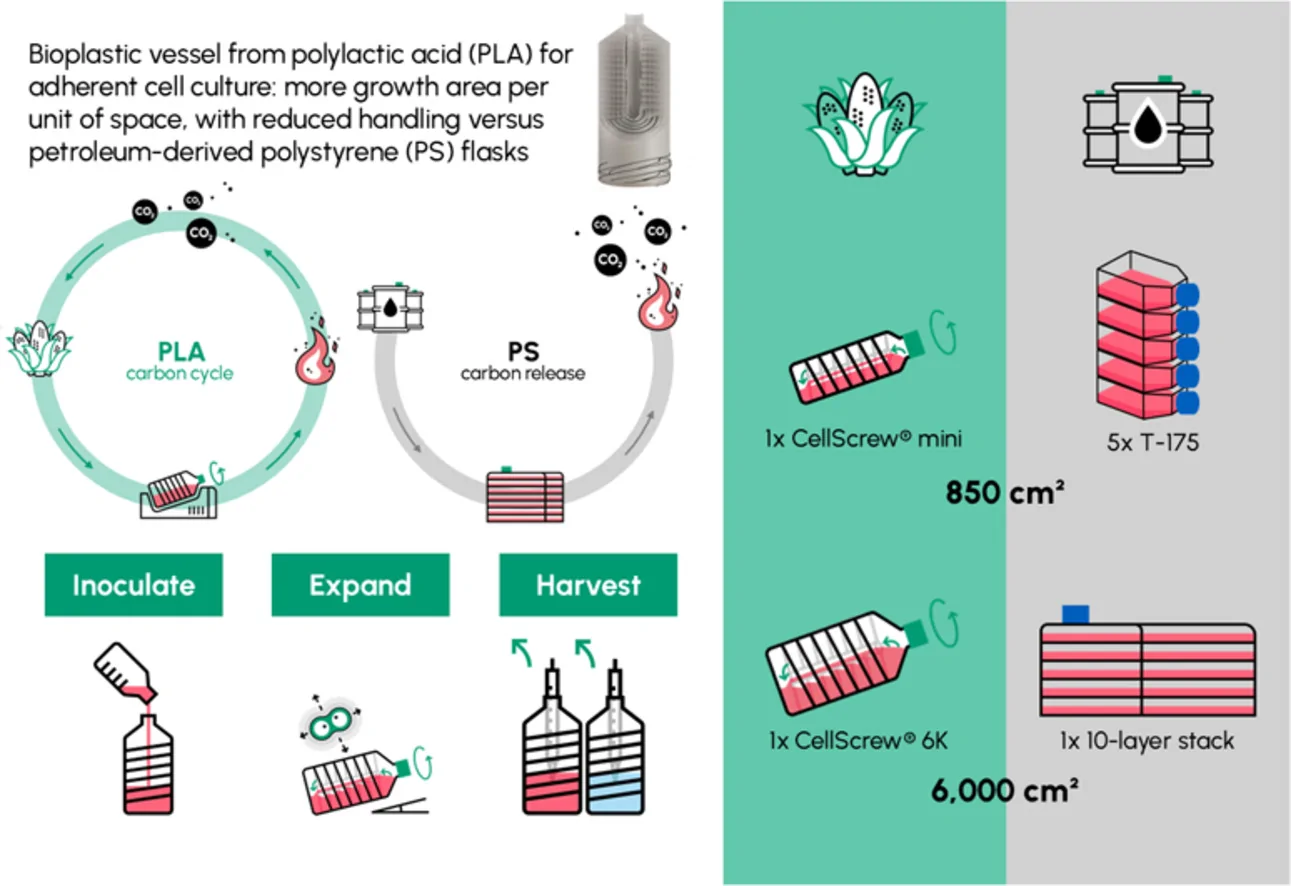

## Specifications table

**Table:** 

**Subject area**	Biochemistry, Genetics and Molecular Biology
**More specific subject area**	Adherent cell culture and bioprocess engineering; adherent mammalian cell expansion
**Name of your method**	Scalable adherent cultivation of CHO-K1 and HEK-293 cells in the CellScrew®
**Name and reference of original method**	None
**Resource availability**	A detailed materials list is provided in the methods section.

## Background

A number of adherent platforms have been developed targeting intermediate-scale cell expansion for research and bioproduction applications, differing in cost, handling, and scalability. These include parallelized T-flasks, multilayer polystyrene stacks, fixed-bed or hollow-fiber reactors, and microcarrier suspensions in stirred-tank reactors.

Parallelized T-flasks remain a prevalent platform in academic and small-scale experimental settings due to their simplicity and low cost. However, operator workload, incubator volume demand, contamination risk, and single-use plastic waste all scale linearly with vessel number [1]. Multilayer stacks address this limitation by consolidating 10–40 trays in a single housing thereby improving space efficiency. Yet they remain ergonomically demanding, are constrained by gas exchange and represent scale-out rather than scale-up [1]. Improved gas exchange is instead offered by fixed-bed and hollow-fiber reactors which enable high-density, perfusion-based continuous operation and are widely used in vaccine and viral-vector manufacturing, with commercial systems scaling to growth surfaces of up to ∼500 m² [2]. Nevertheless, their embedded growth matrix limits real-time monitoring and impairs efficient, high-viability cell harvest, making these platforms poorly suited for seed-train applications where iterative recovery and reseeding of viable cells is required. Microcarrier suspensions in stirred-tank reactors decouple surface area from vessel count and are well established for large-scale production. This necessitates stirred-tank infrastructure, bead–cell adaptation, and a downstream bead-separation step that can compromise recovery and viability in shear-sensitive cell lines [3].

Across all these systems, petroleum-based materials such as polystyrene remain the standard for lab consumables. Life-science laboratories alone were estimated to generate around 5.5 million tonnes of plastic waste in 2014 [4], with a substantial associated carbon dioxide (CO₂) footprint from production and disposal. In contrast, the bioplastic polylactic acid (PLA) is a polymer derived from renewable resources and is increasingly explored as a more sustainable alternative for disposable laboratory applications [5]. Recent work demonstrated that PLA-based sterile printing can reduce greenhouse gas emissions by up to 80% compared to polystyrene and polycarbonate [6].

The CellScrew® combines this lower-footprint substrate with a geometry designed for single-vessel scale-up. Manufactured from 3D-printed PLA, its concentric-cylinder architecture exposes ~850 cm², ~6,000 cm², or ~10,000 cm² of growth surface depending on the format (the 850 and 6,000 cm² formats are validated here). The internal geometry achieves a high surface-area-to-mass ratio, minimizing material input per cm² of growth surface. Consolidating the capacity of many separate vessels into a single bottle further eliminates the proportional scaling of handling steps, incubator footprint, and plastic waste inherent to T-flask parallelization.

Following the operating principle of conventional roller bottles, the CellScrew® is mounted on a roller device and rotated at 0.5 rpm and a 10 ° angle. During rotation, the Archimedean screw geometry moves the medium through the cylindrical structure, wetting the growth surface with a film of liquid and distributing nutrients and dissolved gases before the medium recirculates through the central tube. Characterization of this system confirmed adequate mass transfer under low-shear conditions, with a volumetric oxygen transfer coefficient (kLa) ranging from 3.03 ± 0.11 h⁻¹ to 7.91 ± 0.17 h⁻¹ and theoretical wall shear rates ranging from 2 s⁻¹ to 22 s⁻¹, depending on rotational speed and CellScrew® size [7]. This principle furthermore decouples medium volume from surface area: only the volume needed to maintain the rotating film is required, reducing medium, reagent, and supplement consumption per unit of growth surface compared with T-flasks or multilayer stacks, thereby lowering both the environmental burden and cost of the upstream process while also enabling routine in-process monitoring.

Plasma treatment renders PLA hydrophilic and cell-adhesive [8]. In the CellScrew®, standard cell lines such as CHO-K1 and HEK-293 attach directly to this surface, without coating, passage or workflow adaptation, at efficiencies comparable to tissue-culture polystyrene [7].

On this basis, we describe a reproducible protocol for expanding adherent mammalian cell lines in the CellScrew®, covering direct inoculation from polystyrene, in-process monitoring, and harvest procedures. The protocol was validated across two cell lines (CHO-K1, HEK-293) and two vessel formats (850 cm², 6,000 cm²), is transferable to other standard adherent lines without prior adaptation and supports applications ranging from intermediate-scale expansion to single-vessel scale-up.

## Method details

### Materials

#### Cell lines

− CHO-K1 (DSMZ ACC-110, RRID:CVCL_0214, Leibniz Institute DSMZ, Braunschweig, Germany).
− HEK-293 (DSMZ ACC-305, RRID:CVCL_0045, Leibniz Institute DSMZ, Braunschweig, Germany).

CellScrew® (Green Elephant Biotech GmbH, Gießen, Germany).

**Table:** 

Product (Cat. No.)	Growth surface	Working volume range
CellScrew® mini (GEB-CS-mini)	851 cm²	50 – 120 mL
CellScrew® 6K (GEB-CS-6K)	6,124 cm²	250 – 800 mL

#### Labware

− 3D-printed custom PLA 6-well plate (Green Elephant Biotech GmbH, Gießen, Germany).
− T175 flasks (83.3912.002; SARSTEDT AG & Co. KG, Nümbrecht, Germany).
− Serological pipettes (SARSTEDT AG & Co. KG, Nümbrecht, Germany).

#### Media, buffer, reagents

− Ham’s F12 (HAM-12-A, Capricorn Scientific GmbH, Ebsdorfergrund, Germany).
− RPMI 1640 (RPMI-A, Capricorn Scientific GmbH, Ebsdorfergrund, Germany).
− Fetal bovine serum (FBS) (P30–3031, PAN-Biotech, Aidenbach, Germany).
− DPBS, w/o: Ca and Mg (P04–36,500, PAN-Biotech, Aidenbach, Germany).
− HEPES (P05–01,500, PAN-Biotech, Aidenbach, Germany).
− (10x) Trypsin 0.5%/EDTA 0.2% in DPBS, w/o: Ca and Mg (P10–024100, PAN-Biotech, Aidenbach, Germany), diluted in DPBS 1:10 for static culture and 1:30 for dynamic harvest in the CellScrew®.
− Freezing Medium (P07–90050, PAN-Biotech, Aidenbach, Germany); storage at −80 °C or in liquid nitrogen (−196 °C).
− Erythrosin B (0331, Carl Roth GmbH + Co. KG, Karlsruhe, Germany).
− Green CMFDA (HY-126,561, MedChemExpress LLC, Monmouth Junction, NJ, USA).

#### Equipment

− Roller device (88,881,004, Thermo Fisher Scientific Inc.), set to 0.5 rpm at customized 10 ° inclination.
− Humidified incubator (HERAcell™ 150, Thermo Fisher Scientific Inc.), set to 37 °C and 5 % CO_2_.
− JuLI Smart Fluorescent Cell Viewer (NanoEnTek Inc., Hwaseong-si, Republic of Korea).
− BIOSEN C-line glucose/lactate analyzer (EKF-diagnostic GmbH, Barleben, Germany).
− Rubber mallet (2932–680, CCLIFE).

### Cell culture

CHO-K1 and HEK-293 cells were obtained from the DSMZ (CHO-K1: ACC-110, HEK-293: ACC-305) and cultured in polystyrene T175 flasks with culture conditions specified in Table 1. Cells were passaged twice weekly, at three- to four-day intervals upon reaching a confluence of approximately 90 %. Monolayers were washed with 25 mL (0.14 mL/cm²) DPBS and dissociated with 3 mL (0.017 mL/cm², ≈8.5 µg trypsin/cm²) trypsin/EDTA for 5 min at 37 °C, after which the enzymatic reaction was stopped by addition of 7 mL of the respective FBS-containing culture medium. Cell concentration and viability were determined by staining a 0.2 mL aliquot of the five-fold diluted cell suspension with Erythrosin B and counting four squares of the Neubauer chamber. Cells were then reseeded at the densities specified in Table 1 according to the vendor’s recommendations. Working volumes were selected individually for each cell line based on their specific requirements, determined prior to the seed train.

**Table 1:** Culture conditions of CHO-K1 and HEK-293 cells. FBS concentrations are expressed as % (v/v).

Cell Line	CHO-K1	HEK-293
Culture medium for T175 flask	25 mL Ham’s F12 with 10% FBS	60 mL RPMI 1640 with 10 % FBS and a final concentration of 25 mM HEPES
Seeding density	4,000 cells/cm²	66,000 cells/cm²
Dissociation reagent	Trypsin (0.5 g/L) / EDTA (0.2 g/L)	Trypsin (0.5 g/L) / EDTA (0.2 g/L)

### Workflow for handling the CellScrew® mini with CHO-K1

Four T175 flasks were seeded at 4,000 cells/cm² as a seed train four days before inoculation of the CellScrew® minis. At >95 % confluence, determined by visual inspection using a light microscope, the medium was aspirated, and the cells were washed with 25 mL (0.14 mL/cm²) DPBS. The cells were dissociated by adding 3 mL of trypsin/EDTA (0.017 mL/cm², 8.5 µg trypsin/cm²) and incubated for 5 min at 37 °C. Complete dissociation was verified by light microscopy, and 7 mL Ham’s F12 growth medium was added to neutralize trypsin activity. The cell suspensions of all four flasks were pooled and centrifuged for 5 min at 400 × g. The pellet was resuspended in 40 mL of Ham’s F12 growth medium, and cell concentration was determined as described above.

To inoculate the CellScrew® mini at a seeding density of 4,000 cells/cm², the cell suspension was diluted in prewarmed Ham’s F12 growth medium to a final volume of 85 mL (0.10 mL/cm²) and transferred into the vessel with a serological pipette. To ensure uniform wetting of the internal growth surface, the vessel was held at a 10° angle and rotated manually in the clockwise direction until the medium had covered the entire growth surface, a process that took approximately one minute with gentle rotation. The vessel was then placed onto a roller device set to 0.5 rpm at a 10 ° inclination inside a humidified incubator (37 °C, 5 % CO₂). For the 7-day cultures, a single complete medium exchange was performed at 96 h (day 4). In total, 21 CellScrew® minis were inoculated from the same seed train.

Every 24 h, three CellScrew® minis were harvested independently as biological replicates. First, a 1 mL supernatant sample was withdrawn using a serological pipette and analyzed for glucose and lactate on a BIOSEN C-line analyzer (EKF-diagnostic GmbH, Barleben, Germany) according to the manufacturer’s instructions. The remaining medium was aspirated, and the growth surface was rinsed with 50 mL (0.059 mL/cm²) DPBS by rotating the vessel manually clockwise at a 10 ° angle. After discarding the DPBS, cells were detached with 50 mL of diluted trypsin/EDTA (0.059 mL/cm², ≈10 µg trypsin/cm²) in DPBS on the roller device for 15 min at 0.5 rpm at 37 °C. The detachment time was extended to 15 min (versus 5 min for static T175 cultures) because the larger volume needed to wet the rotating surface, combined with the lower trypsin concentration, required more time to uniformly loosen the attachment complexes throughout the vessel before mechanical force was applied. Surface-protein integrity following this extended exposure was not specifically assayed and should be assessed when the protocol is applied to other, potentially more sensitive, cell types.

Trypsin was used at two working strengths, 0.5 g/L for static T175 passaging and a three-fold dilution to 0.17 g/L for dynamic harvest in the CellScrew®, since the larger volume required to wet the rotating growth surface kept the enzyme dose per unit area within the same range as static passaging (≈8–10 µg trypsin/cm²). This gentle, extended-contact detachment achieved a harvest efficiency of 94 ± 1 % at >93 % viability. The reaction was stopped by addition of 50 mL Ham’s F12 growth medium. Subsequently, the vessel was removed from the roller device, and residual attached cells were dislodged by tapping uniformly along the entire length of the bottle with soft mallet under continuous manual rotation, applying force for 30 - 60 s.

The cell suspension was collected using a serological pipette, and the concentration and viability of this primary harvest fraction were determined as described above. To quantify residual cells on the growth surface, the CellScrew® mini was rinsed with a second 50 mL (0.059 mL/cm²) DPBS volume and tapped again under accelerated manual rotation and even mallet force for approximately one minute. This secondary suspension was collected and counted separately. Harvest efficiency was calculated as the ratio of cells recovered in the primary fraction to the sum of the primary and secondary fractions.

On day 4 post-inoculation, cells harvested from the CellScrew® minis were cryopreserved. From each vessel, cells were resuspended in Freezing Medium at 500,000 cells/mL and dispensed at 1 mL per cryovial. Following controlled-rate cooling at 1 °C/min, vials were stored for 7 days either at −80 °C or in liquid nitrogen (−196 °C). After storage, cells were thawed and seeded into separate T175 flasks and cell density was monitored over the first two post-thaw passages.

### Workflow for handling the CellScrew® 6K with HEK-293

HEK-293 expansion in the CellScrew® 6K was characterized across five biologically independent experiments (n = 5), each consisting of an independent seed train and a separately inoculated CellScrew® 6K vessel. For each biological replicate, a seed train of eight T175 flasks (one CellScrew® 6K inoculated from eight T175 flasks) was established four days prior to inoculation. Once the desired confluence was reached, the seed train T175 flasks were harvested. Medium was aspirated, each flask was washed with 25 mL (0.14 mL/cm²) DPBS, and cells were detached with 5 mL of trypsin/EDTA (0.029 mL/cm², ≈14.5 µg trypsin/cm²) for 5 min at 37 °C. The reaction was stopped with 5 mL RPMI 1640 growth medium, and the cell suspensions were pooled into 50 mL conical tubes. The cell concentration of the pooled suspension was determined using a Neubauer chamber.

Each CellScrew® 6K was then inoculated at 66,000 cells/cm² in a working volume of 600 mL (0.098 mL/cm²) and cultivated under the conditions described above to a defined endpoint of 7 days. This endpoint was predetermined by preceding studies in a smaller scale. Glucose and lactate concentrations in the culture supernatant were measured every 24 h post-inoculation. The first complete medium exchange was performed 48 h after inoculation and daily thereafter.

To characterize early attachment kinetics, one additional CellScrew® 6K was inoculated at a higher density of 78,380 cells/cm² (resulting in 800,000 cells/mL), and 1 mL supernatant samples were taken hourly during the first 6 h and again at 24 h post-inoculation, with the concentration of non-attached cells determined in each sample. The attached fraction was calculated as the inoculum actually used minus the non-attached cells recovered in the supernatant, expressed as a percentage of that inoculum. Any proliferation within the non-attached fraction would therefore lower rather than inflate this value, making the reported percentages a conservative, minimum estimate of attachment.

At the scheduled endpoint, the medium was aspirated, and the growth surface was washed with 300 mL (0.049 mL/cm²) DPBS by rotating the CellScrew® 6K clockwise at an angle of 10 °. After discarding the DPBS, cells were detached with 300 mL of diluted trypsin/EDTA (0.049 mL/cm², ≈8.3 µg trypsin/cm²) in DPBS on the roller device at 0.5 rpm for 10 min. The detachment time was extended to 10 min (versus 5 min for static T175 cultures) because of parallel reasons as described above for the CellScrew® mini. Since HEK-293 cells detach more readily than CHO-K1, trypsin incubation was extended less than for the CHO-K1 experiments.

The vessel was then transferred from the roller device to a benchtop, and residual attached cells were dislodged by uniformly tapping with a soft mallet under continuous manual rotation. The resulting trypsin/cell suspension was collected using a serological pipette, the growth surface was rinsed with a second 300 mL of DPBS under manual rotation, and this wash fraction was combined with the primary suspension to yield a pooled harvest. The cell concentration of the pooled harvest was determined as described above, and the endpoint cell density was calculated by normalizing the total cell number to the growth surface of 6,124 cm² (theoretical surface area derived from the CAD model).

## Method validation

### Growth, metabolism and harvest efficiency of CHO-K1 cells, cultured in the CellScrew® mini

Unless otherwise stated, ± values reported throughout this section denote mean ± standard deviation (SD), not the standard error of the mean (SEM). CHO-K1 cells were cultivated adherently in CellScrew® mini (851 cm² growth surface, 85 mL working volume) as described in Section “Workflow for handling the CellScrew® mini with CHO-K1” and Table 1.

**Fig. 1:**
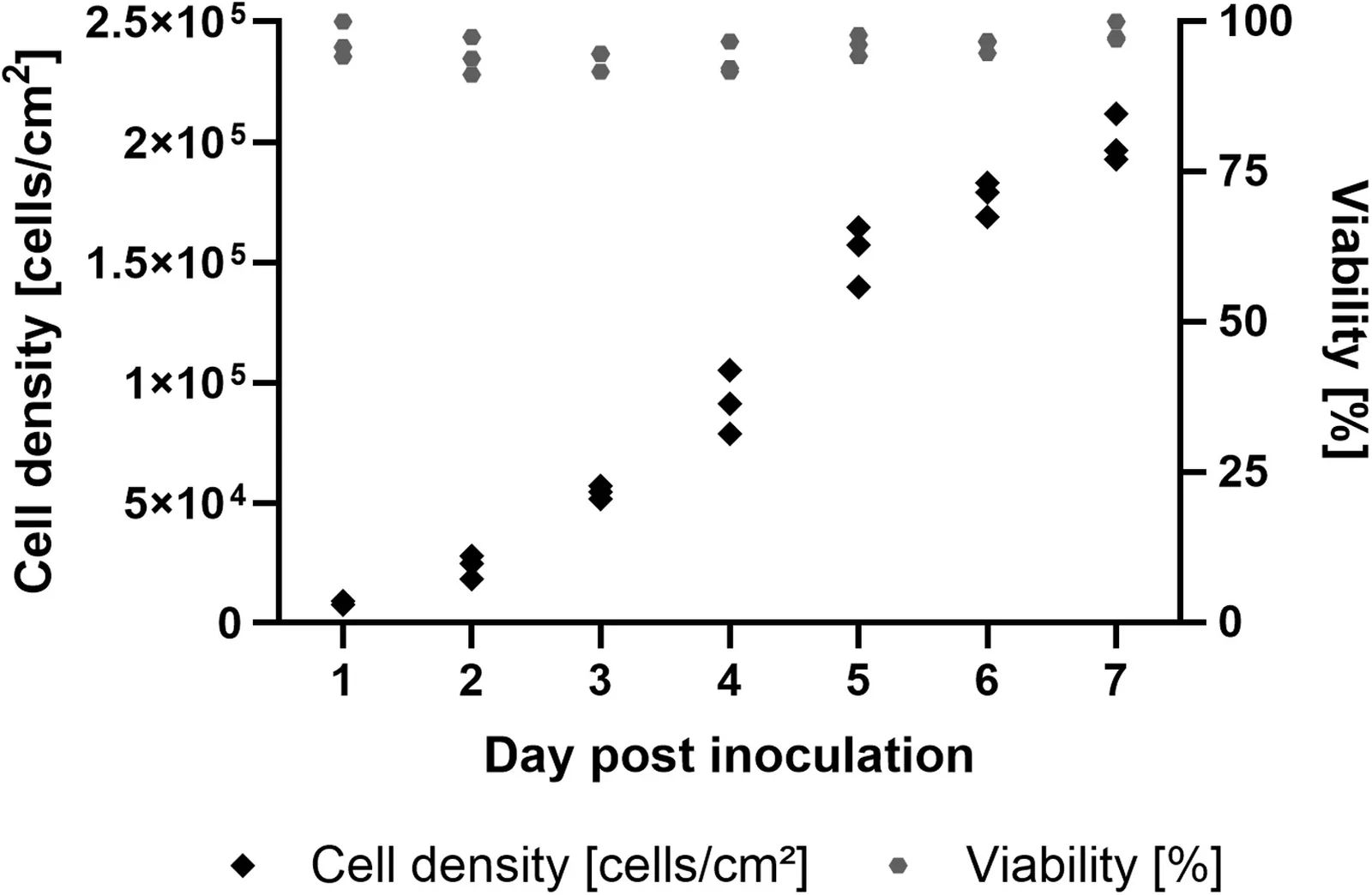
CHO-K1 cell density and viability in the CellScrew® mini across 7 days of cultivation (n = 3 biological replicates from a single seed train). Cells expand approximately 50-fold from the seeding density of 4,000 cells/cm² to 200,000 cells/cm² on day 7, with an average doubling time of 21.5 h between days 1 and 5, while viability remains above 93% on every day.

**Fig. 2:**
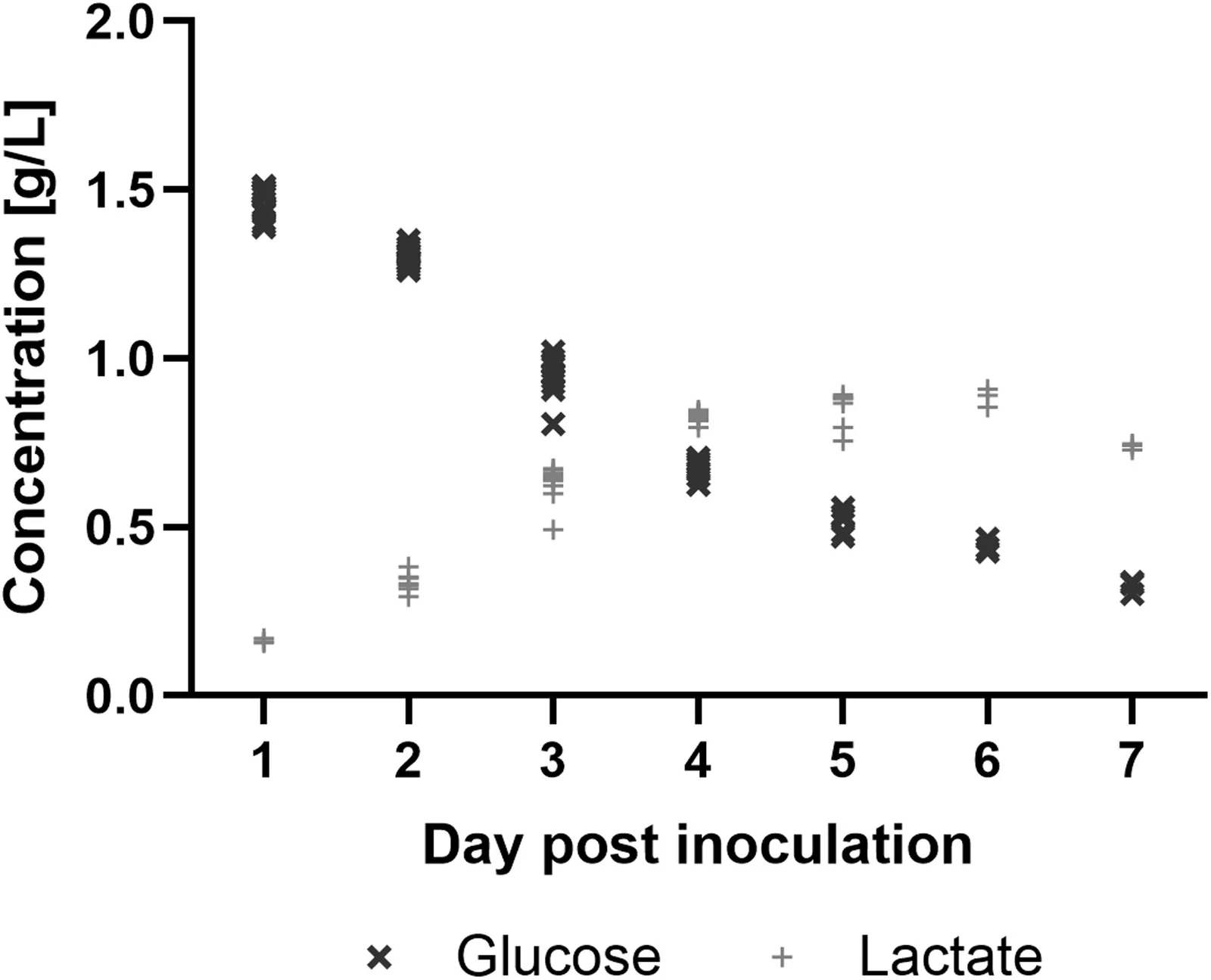
Glucose consumption and lactate accumulation in CHO-K1 cultures in the CellScrew® mini over 7 days (n = 3 biological replicates from a single seed train, supernatant samples were taken daily from all available CellScrew® mini in a range from 3 ). Glucose decreases steadily from 1.46 to 0.32 g/L while lactate accumulates to a peak of 0.89 g/L, demonstrating stable metabolic activity of the cultured cells.

Seeded at 4,000 cells/cm², the cells expanded consistently over seven days, reaching a final density of 200,000 ± 10,000 cells/cm² on day 7, corresponding to a roughly 50-fold expansion relative to the inoculation density (Fig. 1). Apparent doubling times tₙ, calculated from consecutive cell densities N₁ and N₂ over a time interval Δt as $t_{n}=\Delta t\times \frac{ln(2)}{ln(N_{2}/N_{1})}$, averaged 21.5 h between days 1 and 5, consistent with exponential growth. Glucose consumption and lactate formation followed the expected metabolic profile: glucose decreased steadily from 1.46 g/L on day 1 to 0.32 g/L on day 7, while lactate accumulated in parallel, peaked at 0.89 g/L on day 6, and then declined slightly to 0.74 g/L on day 7 as glucose was progressively consumed (Fig. 2). This modest change is consistent with lactate re-uptake at low glucose [9], although assay variability cannot be excluded. The apparent lactate-to-glucose yield over the culture was ≈1.6 mol/mol (0.89 g/L lactate produced per 1.14 g/L glucose consumed, ≈0.78 g/g), below the theoretical maximum of 2 mol/mol and consistent with the predominantly glycolytic metabolism typical of CHO-K1 in batch culture [9].

Alongside the cell densities and metabolic profile, the harvest efficiency and culture health were monitored. Averaged across days 2–6 (days 1 and 7 were not included because the purified primary fraction was lost during handling by a different operator, leaving only the pooled cell density available for these two days), harvest efficiency under the reduced trypsin/EDTA concentration and mechanical-tapping protocol (Section "Workflow for handling the CellScrew® mini with CHO-K1") was 94 ± 1%, confirming that cell recovery was consistent across the exponential growth phase and independent of culture density. Cell viability remained above 93% on all days, confirming that neither the expansion in the CellScrew® nor the harvest procedure compromised culture health.

### Cryopreservation and post-thaw recovery of CHO-K1 cells, previously cultured in the CellScrew® mini

**Fig. 3:**
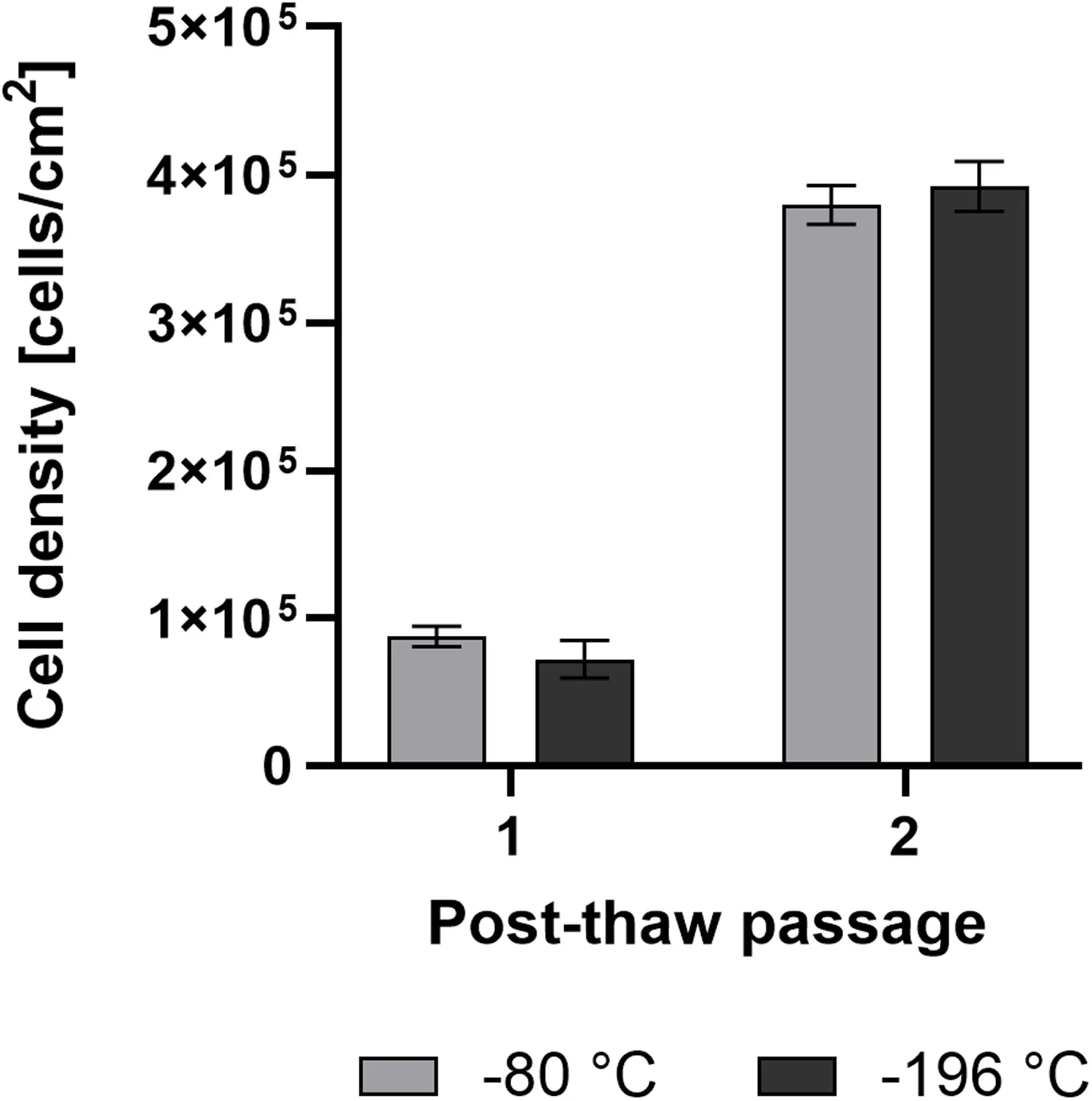
Post-thaw recovery of CHO-K1 cells cryopreserved at −80 °C or in liquid nitrogen, reseeded in T175 flasks and counted at the end of passages 1 and 2 (n = 3 from independent thaw and passage procedures, mean ± SD). Both storage conditions reach comparable densities at every passage and recover to 379,000 cells/cm² (−80 °C) and 392,000 cells/cm² (liquid nitrogen) by passage 2 — approximately 3-fold above the 125,000 cells/cm² DSMZ reference density [10].

To confirm that cells cultivated adherently on the PLA surface of the CellScrew® retain standard cryopreservation and post-thaw behavior, cells harvested from the CellScrew® mini were cryopreserved and subsequently thawed and re-expanded in conventional T175 flasks following the standard passaging procedure. Post-thaw recovery was benchmarked against the DSMZ-reported 125,000 cells/cm² for CHO-K1 (ACC-110) [10]. Cells were stored either at −80 °C or in liquid nitrogen (−196 °C) prior to thawing. After the first post-thaw passage (P1), both storage conditions reached a comparable recovery, with cultures stored at −80 °C reaching 87,500 cells/cm² (± 7,000; ∼70% of the reference density) and cultures from liquid nitrogen reaching 72,000 cells/cm² (± 12,700; ∼58% of the reference density). By the second post-thaw passage (P2), cultures from both conditions exceeded the reference density, reaching 379,000 cells/cm² (± 13,200) and 392,000 cells/cm² (± 16,900), respectively. These results demonstrate that CHO-K1 cells expanded on PLA in the CellScrew® can be cryopreserved, thawed, and re-expanded using standard laboratory workflows without loss of downstream viability or growth performance, as the two tested storage conditions yielded comparable outcomes within two passages (Fig. 3).

### Attachment and expansion of HEK-293 in the CellScrew® 6K

**Fig. 4:**
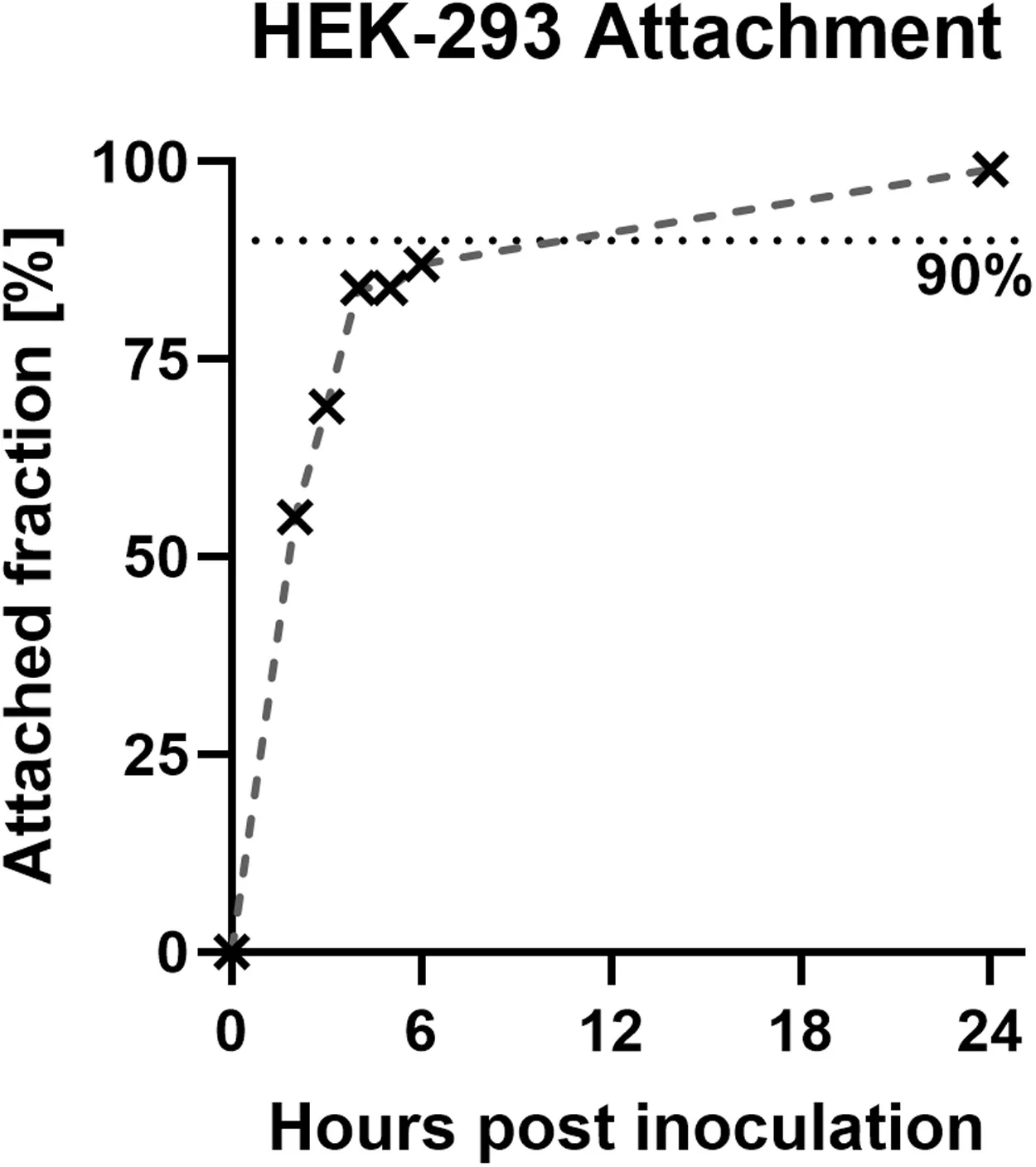
Attached fraction of HEK-293 cells on the CellScrew® 6 K growth surface over the first 24 h post inoculation. The bulk of attachment occurs within the first four hours and is essentially complete by 24 h, indicating that the PLA surface supports rapid HEK-293 adhesion.

To resolve early attachment kinetics, a CellScrew® 6K was inoculated at a density of 78,380 cells/cm² (resulting in 800,000 cells/mL), and the non-attached cell fraction in the supernatant was sampled hourly from 2 to 6 h and again at 24 h post-inoculation, and calculated against the original inoculum concentration. The attached fraction increased from 55% at 2 h to 69% at 3 h and 84% at 4 h, then remained essentially stable through 6 h (84% at 5 h, 87% at 6 h), and reached 99% by 24 h. These data indicate that the bulk of attachment to the PLA growth surface occurs within the first four hours under continuous rotation at 0.5 rpm, followed by a slow terminal phase between 6 and 24 h (Fig. 4).

**Fig. 5:**
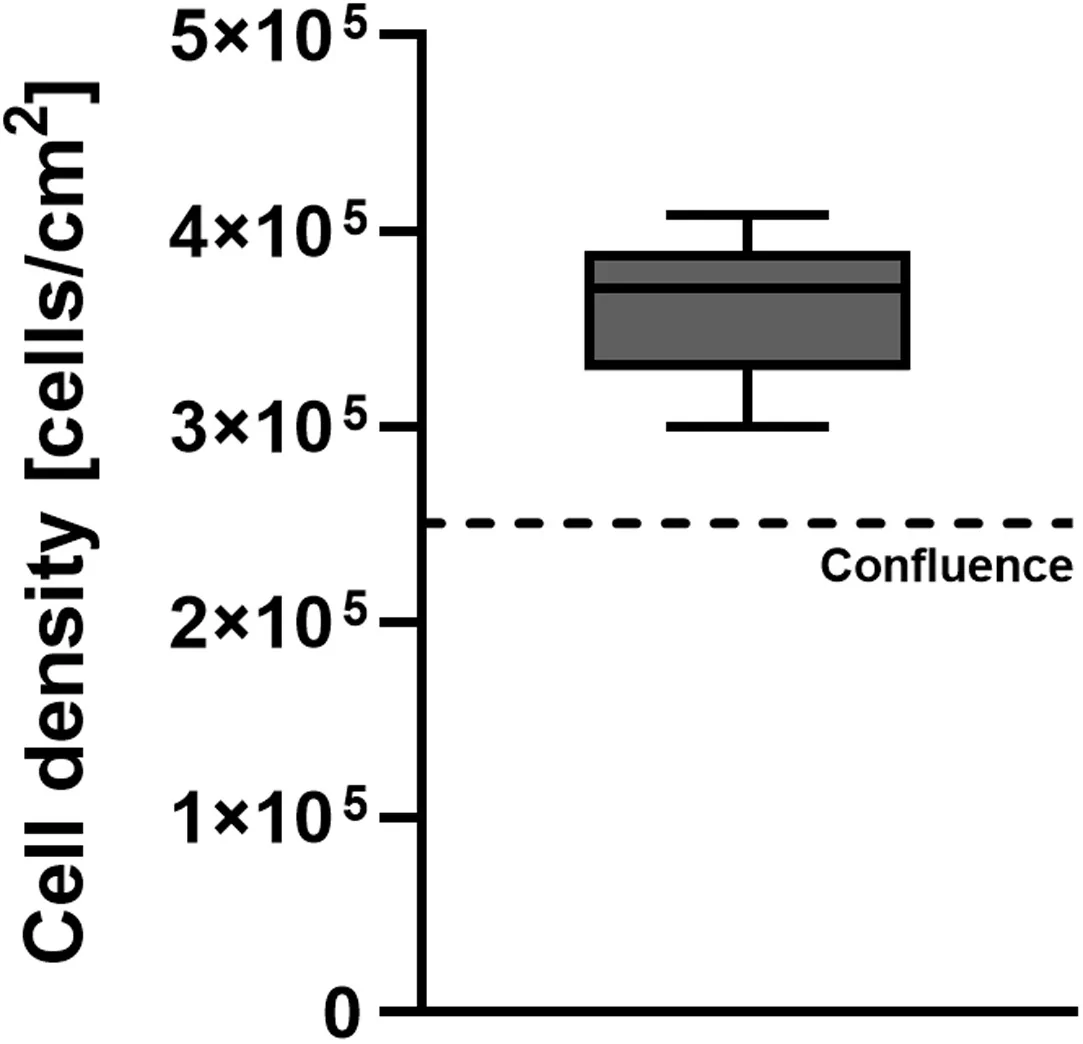
Endpoint cell density of HEK-293 cultures in the CellScrew® 6K (n = 5). Each CellScrew® 6K was inoculated from an independent seed train of eight T175 flasks at 66,000 cells/cm² and harvested at the standard endpoint. Mean endpoint density was 361,000 cells/cm² (CV 10.9%), demonstrating reproducible expansion performance across all five runs.

Following this attachment phase, the cells expanded reproducibly to a high endpoint density. Across five biologically independent experiments, the mean endpoint cell density was 361,000 cells/cm² (SD 39,200; CV 10.9%). During the study, the FBS lot was changed, a recognized source of run-to-run variability that should be considered when interpreting the growth data. Nonetheless, expansion performance remained within a narrow band across all five independent runs (Fig. 5). Notably, all individual endpoint densities exceeded the manufacturer's reference expectation of 250,000 cells/cm², confirming that the CellScrew® 6K supports expansion outcomes consistent with established cell line performance standards. Because these endpoint densities exceed single-monolayer confluence, the cells likely grew in multiple layers. The possible consequences, including contact inhibition, nutrient and gas supply, and long-term genomic stability, were not examined here and warrant further study.

### Visualization of the spatial distribution of adherent cells on PLA surfaces

**Fig. 6:**
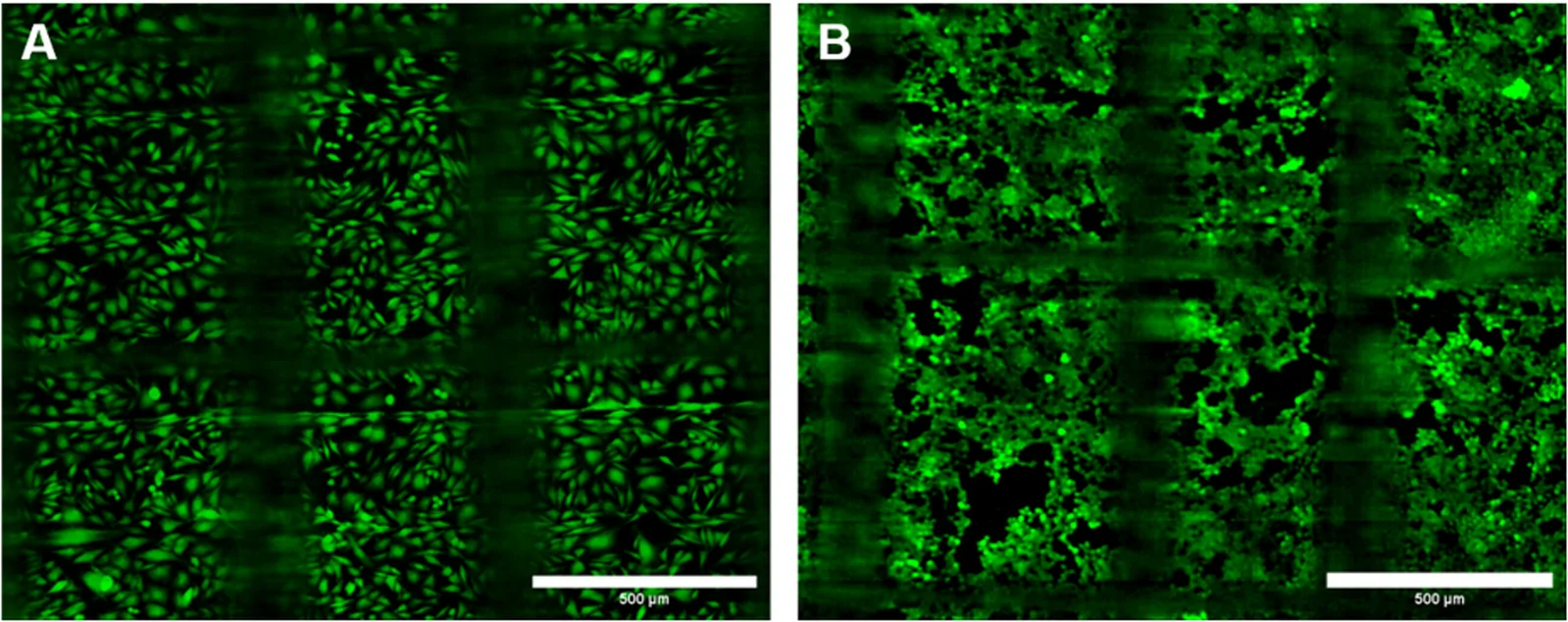
CMFDA-stained CHO-K1 (A) and HEK-293 (B) cells on the 3D-printed PLA growth surface, cultured statically in PLA well-plates of the same material used in the CellScrew®. Both cell lines show uniform coverage, confirming that the PLA substrate supports even adherent growth (scale bar, 500 µm).

To visualize the spatial distribution and overall morphology of the growing cells, both lines were cultivated on the same PLA material but in a static 6-well format, allowing subsequent microscopic analysis. Living cultures were stained with Green CMFDA (5 µM for 5 min), revealing even distribution and growth throughout the 3D-printed surface (Fig. 6). As this static 6-well format is non-rotating and not film-wetted, it does not mimic CellScrew® hydrodynamics and is intended only to demonstrate the general feasibility of the 3D-printed PLA surface for adherent growth.

Overall, these protocols establish the CellScrew® as a lower-footprint platform for intermediate-scale adherent culture, delivering cellular performance comparable to polystyrene-based culture. Replacing polystyrene with PLA [5,6], concentrating the growth surface into a single vessel, and leveraging the efficient medium economy of the Archimedean architecture [7] reduce both material and operational footprint relative to parallelized formats of comparable surface area. Comparing the system to established adherent culture systems highlights the effect of the volume-to-surface ratio of the CellScrew®. While T-175 flasks (e.g. 25 mL for 175 cm²) require ∼0.143 mL/cm², multilayer stack systems start at ∼ 0.204 mL/cm² (e.g. 1300 mL for 6360 cm² [11,12]), and roller bottles depending on size need ∼ 0.235 mL/cm² (e.g. 200 mL for 850 cm² [13]), whereas microcarrier systems require less, at ∼ 0.076 mL/cm² (e.g. 1,000 mL for 13,200 cm² [14]). The CellScrew® 6 K approaches the lower end of this range with ∼ 0.098 mL/cm² (e.g. 600 mL for 6124 cm²). Since medium, serum, and supplements are direct cost drivers in production processes, optimizing medium consumption can meaningfully lower these costs. Comparing the CellScrew® to the systems mentioned before, media reduction of approximately 31 - 58% are possible. Microcarrier systems remain more medium-efficient but require more extensive process development and downstream processing.

## Limitations

### Protocol transferability

The protocol is established with CHO-K1 and HEK-293 as representative adherent mammalian cell lines, and the same operating principle has been applied to other adherent lines, such as Vero and HeLa, without major changes to the workflow. Only medium volume and exchange intervals were adjusted to match each line’s metabolic profile. Furthermore, based on the data on nutrient supply, gas transfer, and shear rates [7], the system is, in principle, transferable to a broad range of adherent cells, including primary cells, macrophages, mesenchymal stromal cells (MSCs), and induced pluripotent stem cells (iPSCs). However, for each new cell type, seeding density, medium supply, and surface characteristics should be re-evaluated before transfer into the CellScrew® system.

### Surface functionalization

The polylactic acid growth surface is plasma-treated to enhance wettability, supporting attachment and expansion of standard adherent cell lines without coating. More demanding cell types, however, may require surface modification. For instance, iPSCs and MSCs are routinely cultured on defined or ECM-based coatings on static polystyrene, so surface modification would be expected on the CellScrew® as well [15,16]. Recombinant protein coatings and Matrigel®, the latter an undefined, xenogeneic basement-membrane extract rich in extracellular-matrix proteins [16], are being evaluated on the vessel surface in ongoing in-house work. Protocols established for static surfaces will likely need adaptation to the rotating geometry, where continuous rotation and intermittent wetting can compromise uniform deposition. Establishing the appropriate surface conditions for each new cell type, ranging from no modification to a dedicated coating, is therefore the first parameter to address on this platform.

### In-process monitoring

The opaque PLA structure precludes direct microscopic inspection of the attached cell layer during a run, so confluence and morphology cannot be assessed visually, and the yield cannot be measured directly during a run. It must either be obtained by sacrificial harvest or inferred from indirect parameters such as glucose/lactate kinetics, which first need to be calibrated for each new cell type in small-scale pilot runs. While visual confirmation of growth remains common practice, visual readouts are highly setup-dependent and difficult to objectify. Quantitative measurements, in particular supernatant metabolites such as glucose and lactate, provide a more reliable and reproducible picture of the culture state and support operator-independent, scalable workflows [9]. Supernatant glucose and lactate concentrations have proven to be a reliable and simple readout for monitoring growth as they require only a small sample of medium and can be measured on standard bench-top analyzers. Because accumulating lactate acidifies the medium, culture pH can serve as a further low-effort proxy for metabolic activity alongside glucose and lactate.

### Outlook

Looking beyond the cell lines and formats validated here, the platform offers clear potential to scale.

Given its low shear-stress profile and the successful expansion of HEK-293 cells demonstrated here, the CellScrew® may be suitable as a small-scale production vessel for virus and viral vector manufacturing. Other anchorage-dependent lines commonly used in this context, such as Vero cells, could similarly benefit from the platform's gentle rotational culture format, though this remains to be experimentally confirmed. The stepwise, high-frequency medium exchange regime already implemented here as daily medium changes, resembles a semi-continuous process and could be extended toward a perfusion system by adding a pump and controller system to the CellScrew®, enabling continuous product harvest without disrupting the rotating culture format. Green Elephant Biotech GmbH is working on an automation platform which is published (Patent: EP4596667A1; WO2025162880A1). Because the CellScrew®’s production relies on additive manufacturing, the achievable growth surface is limited only by the printer's build volume and can be extended to larger formats if needed.

On the monitoring side, the supernatant readouts used here rely on manual sampling today but translate directly into in-line sensing, for instance realized via Raman spectroscopy for glucose and lactate monitoring or optical dissolved-oxygen sensors, once a closed-system adapter is integrated, enabling continuous, real-time process control. An initial in-house, cradle-to-grave product carbon footprint estimates approximately 5.5 kg CO₂ equivalents per CellScrew® 10K (10,000 cm² surface) versus 38.3 kg CO₂ equivalents for a polystyrene multilayer stack of equivalent surface area, an approximately 86% reduction based on ecoinvent and GEMIS emission factors and driven largely by the plant-based PLA raw material [17]. As preliminary internal data that exclude the use phase and have not undergone independent critical review, these figures should be interpreted with caution. An externally reviewed, multi-category life-cycle assessment including the use phase is a natural next step. Taken together, the CellScrew® demonstrates that adherent cell expansion can be decoupled from petroleum-based single-use plastics without compromising performance, representing a tangible step toward more sustainable bioprocessing at scale.

## Ethics statements

Not applicable.

## Declaration of generative AI and AI-assisted technologies in the manuscript preparation process

During the preparation of this work the author(s) used Claude (Anthropic) in order to assist with language and structural editing. After using this tool, the author(s) reviewed and edited the content as needed and take full responsibility for the content of the published article.

## CRediT authorship contribution statement

**Fabian Watzl:** Writing – original draft, Data curation, Formal analysis, Visualization. **Johanna Eichberg:** Investigation. **Annika Herchenröder:** Investigation. **Katharina Günther:** Conceptualization, Writing – review & editing, Funding acquisition. **Lukas Käßer:** Conceptualization, Supervision, Project administration, Writing – review & editing. **Björn Boshof:** Investigation, Supervision, Writing – review & editing.

## Declaration of interests

The authors declare the following financial interests/personal relationships which may be considered as potential competing interests: All authors are employees of Green Elephant Biotech GmbH, the manufacturer of the CellScrew® described in this article.

## Data Availability

Data will be made available on request.
